# Notices

**Published:** 1867-05

**Authors:** 


### NOTICES.

The American Tract Society, 150 Nassau street, New York, have issued "Lile Ada," reprinted from a London edition, also "Sun Beams for Human Hearts," from God's own word, both sent post-paid for 12 cents. Also, "A Mother's Legacy," or Sabbath evening counsels to her sons and daughters, by Mrs. Nancy Sproat, late of Taunton, Mass., author of Poetic Works for the Young, &c. Mrs. Sproat was an earnest and devoted Christian mother, and this little twenty-five cent book will be a great aid to young mothers as well as to growing children. "The Bible Reader's Help," 30c., with painted maps of the Holy Land and of Bible countries, contains valuable and interesting articles on the books of the Bible; its doctrines and teachings, the English version; its figurative language, reading hints; the patriarchs; the parables; the miracles; Bible places, words, images, customs, &c., explained; just such a book as will aid in attaining the truthful meaning of that book, which is worth more than all other books beside; a book of which some one has beautifully written,

> 'This little book I'd rather own
> Than all the gold and gems,
> That e'er in monarch's coffers shone,
> Or all their diadems.
> Yes, were the seas one chrysolite,
> The earth one golden ball,
> And diamonds too the stars of night,
> This book were worth them all."

"Toils and Triumphs," of 25 years, by one of the Secretaries of the American Tract Society, describing the colportage system, its aims, needs, effects, &c.

"Hints and Thoughts," for Christians, by Rev. John Todd, D. D., sent for one dollar, in beautiful, clear, large type, may be profitably read in every Christian family; it is practical, comforting, suggesting, suitable for a summer traveller; can be taken up and read for a moment or two, then laid down and reflected upon, as the subjects are varied and numerous and attractive.

"Back Bone." 396 pp. Robert M. DeWitt, 13 Frankfort street, publisher. Author, Edward H. Dixon, M. D., editor of "The Scalpel," dedicated to "Horace Greeley and Peter Cooper, friends of their country and of mankind." This book is made up of articles which originally appeared in "The Scalpel." It is altogether a slashing production, consequently titillates a few, makes many enemies, and injures the author more than any one else. Dr. Dixon is nothing, if not severe; right soundly does he thrash the follies of the times, and its stupidities; and at every page, the reader is obliged to cry out, "Hurrah for Dixon, its all true;" and yet, that same reader would not like to adopt that plan of reforming the world, nor would he like to be seen patting the author on the back encouragingly; just as a good many people would like hugely to see some bad citizen kicked, but would not dare to be the kickers. Dixon would rather lay on the blows, than see it done, because he would be afraid it would not be done with merited vigor. The book is full of instruction in matters pertaining to physical, mental and social health. Dr. Dixon is a scientific man, and his physioligical views will be found to bear a rigid examination, for he knows whereof he affirms. As to matters of morals and religion, he is not a safe guide ; with this caveat, the intelligent reader will be instructed and amused. But if any one wants a limb amputated or a head taken off, be assured it will be done secundem artem, by Edward H. Dixon, Surgeon, at 45 Fifth Avenue, New York.

If any of our subscribers do not receive their Journal, they will be re-sent on notification, as a matter of courtesy, but not of right, as all his responsibility ceases, the moment a publisher deposits his publication in Uncle Sam's mail bag. Whoever claims a second number of the Journal, as a matter of right, won't get it. Whoever is fool enough to write to the publisher while he is in a passion is not considered worth a three cent postage stamp and an old envelope turned inside out. And whoever wants an answer to any letter on any subject must enclose a post-paid envelope already addressed, as a very large number of persons cannot write their own names legibly or fail to give the full post-office address of town, county and state. We frequently receive letters containing from two to twenty dollars, without date or name or place; sometimes they reach us through the dead letter office at Washington, for want of a postage stamp, or put on so loosely as to fall off. In any company of ten persons, there will scarcely be found three who know how to attach a stamp to a letter properly. The place to attach a stamp is the upper right hand corner; the manner is more important; very many begin by licking the gum off the stamp; if they knew how dirty it was perhaps they would'nt do it; but what's the use of licking off the gum, there is scarcely enough put on it any time to make it stick well. Moisten the envelope itself with the saliva, and then press the stamp on it with the finger; or better, a clean handkerchief. Reader, put it to yourself, have you been one of the lickers of dirty gum; if you have, you are one of that large class which has very little common sense, not enough we fear to keep you from killing yourself one of these days by some thoughtless act, which will prove that for the time being, at least, you were scarcely one remove from an idiot. Havn't you done a thing before now, in reference to which, when afterward thinking of it, you exclaimed with gritted teeth and ungovernable impatience, "what a fool I was." This being so you are the very person, who upon your clothing taking fire, would run to the door with all your might, fanning the flame, and allowing it to burn upwards around your face, drawing it into the throat and lungs, causing certain death, instead of lying down on the floor, thus preventing any flame being about the face, and then drawing a carpet to you, or a piece of bedding, and rolling yourself up in it, which would instantly extinguish the flame; or you are one of that other kind, who instead of tasting a thing in an unmarked bottle or covered vessel, gulp it down at a draught, it being cyanic acid, or other deadly poison, killing you in an instant; as thousands and tens of thousands have done; or if waking up in the night, and finding your room on fire, you would jump up and run to the door or window to be suffocated with smoke and flame, instead of falling on the floor and crawling out ; the smoke and flame and heat being above you; if your carriage was running away, you would jump out at the side in double quick time, and break your invaluable neck, instead of spilling yourself out from behind, and let the carriage run until it was tired; if wearied and overheated with walking, you would take a stage or carriage or omnibus, let down a window and breathe in the delicious cool clear air, to wake up with inflammation of the lungs next morning, and in three days be in your grave, instead of closing the window if it was open, and drawing your clothing closer about you, so as not to cool off too quickly; if you had a foot frozen in skating, or by long exposure to very cold weather, you would as soon as you get home, ram it in a pail of hot water, and then have a surgeon to saw it off in a few days, instead of putting it in ice cold water first, and allow it to be gradually warmed; instead of eating to your heart's content, like any other pig and then stop, you continue eating against your instinct, merely because you don't like to leave a lump of butter or a bit of bread or a piece of meat, and that being the last ounce that broke the camel's back, the last crumb that overpowered the laboring stomach, you induce dyspeptia, and for the remainder of life you become an inveterate growler and grumbler, pleased with nothing and nobody, not even with yourself; in fact, so thoroughly disgusted with life, that you are almost persuaded to jump into the river, and would do it, were you not conscious of richly deserving some of the pains and penalties of an after existence. If you are eating, and find that you have had enough, and don't want a mouthful more, why in the world don't you stop, there's not a pig or puppy or poodle in existence that hasn't sense enough to stop eating when he's got enough; your brother the donkey does it, don't you see reader, you are more than a mule ; ain't you ashamed of yourself for doing such stupid things, and putting yourself beneath the brutes which perish. Would you like to be wiser? then without delay purchase a full set of Hall's Journal of Health, bound in muslin, thirteen volumes, for $20, and practice what is therein contained as long as it does you good, and our word for it, you will be a goose no longer, but will become a hale, hearty, healthy, jovial person, full of life and fun, with a heart overflowing with kindliness to all of the human family, just like the editor of Hall's Journal of Health.

